# Effect of Rolling Parameters on Room-Temperature Stretch Formability of Mg–2Zn–0.5Ca Alloy

**DOI:** 10.3390/ma16020612

**Published:** 2023-01-09

**Authors:** Wei Li, Guangjie Huang, Xingpin Chen, Xinde Huang

**Affiliations:** School of Materials Science and Engineering, Chongqing University, Chongqing 400044, China

**Keywords:** magnesium, rolling parameters, microstructure, texture, stretch formability

## Abstract

In this work, Mg–2Zn–0.5Ca (wt.%) alloy sheets fabricated according to various rolling parameters were evaluated to investigate the effect of rolling parameters on room-temperature stretch formability. The sheet rolled at 360 °C with a pass rolling reduction of 10~33% exhibited the worst I.E. value of 4.4 mm, while the sheet rolled at 360 °C with a pass rolling reduction of 20~50% exhibited the best index Erichsen (I.E.) value of 5.9 mm. Among the sheets, the (0002) basal texture intensity was the weakest, and the grain basal poles split away from the normal direction toward both the rolling direction and the transverse direction. Microstructural and deformation mechanism measurements of stretch forming to 2 mm for the sheet rolled at 360 °C with a pass rolling reduction of 20~50% by electron backscatter diffraction and in-grain misorientation axes showed that there was a higher activity of {10–12} extension twins and that a prismatic <a> slip was initiated. As a result, the weakening of the texture and the broader distribution of basal poles in the plane contributed to the improved formability of the sheet rolled at 360 °C with a pass rolling reduction of 20~50%.

## 1. Introduction

Today, as the global energy crisis escalates, the demand for lightweighting is continuing to increase in the automotive and aerospace industries. As the lightest structural metal materials, magnesium and its alloys have great prospects in the fields of automotive, aerospace, and 3C products [1,2,3]. However, the poor plastic deformation ability of magnesium alloys, especially their room-temperature formability, has limited their wide application. The poor formability of magnesium alloys is mainly attributed to their hexagonal close-packed (hcp) crystal structure. Compared to face-centered cubic (fcc) crystal structure metals (e.g., Al), hcp metals have fewer independent slip systems [4]. Moreover, during rolling, magnesium alloy sheets often develop a strong basal texture with a (0002) plane parallel to the rolling plane, which is unfavorable to stretch formability at ambient temperatures [5,6]. Numerous studies have been dedicated to improving the formability of magnesium alloys by weakening the basal texture and microstructure modification [7,8,9,10,11,12,13]. For example, the formability of AZ31 alloy was improved by 41% through a continuous bending process, which weakened the basal texture [6]. Further, AZ31 alloy’s basal texture intensity was reduced from 13.4 m.r.d to 5.2 m.r.d with the addition of Ca to the alloy, resulting in an Erichsen value for AZ31–0.5Ca that was nearly three times higher than that for AZ31 alloy [14]. The fine-grained microstructure enables the prismatic <a> slip to more actively take place in Mg–1.2Al–0.3Ca–0.4Mn–0.3Zn alloy, thus the formability was improved [13]. Therefore, the formability of magnesium alloys can be improved by modifying their textures and microstructures.

Recently, different thermomechanical processing conditions have been shown to be effective in enhancing the formability of magnesium alloys by weakening their textures. The formability of an AZ61 alloy sheet was improved by 1.5 times when the rolling temperature was increased from 450 °C to 520 °C, resulting in a significant decrease in its basal texture intensity [15]. Moreover, the intensity of the basal texture was only 3.1 m.r.d for AZ31 alloy rolled at 550 °C, and its Erichsen value at room temperature was an excellent 9.5 mm [5]. In addition, Shi et al. investigated the effect of different final-pass rolling reduction methods on the microstructure and properties of Mg–1.1Zn–0.76Y–0.56Zr alloy, finding that increasing the final-pass rolling reduction can modify the TD-split texture, achieving the reduction of planar anisotropy. When they increased the final-pass rolling reduction from 30% to 70%, the Erichsen value increased from 7.0 mm to 8.0 mm [16,17]. The reasons for the weakening of texture by thermomechanical processing are complex. In magnesium alloys, the tension twin is easily activated due to the low critical resolved shear stress (CRSS) for activating the tension twin. In addition, in Mg–6Zn–1Y alloys, the basal poles of recrystallized grains in deformation bands composed of tension twins are tilted toward TD, and the basal texture is weakened during rolling [18]. In addition, the activation of the non-basal slip systems in rolling also plays a role in weakening texture [19]. These studies show that using appropriate thermomechanical processing parameters during rolling is a feasible approach for enhancing the formability of magnesium alloys.

In Mg–Zn–Ca alloys, researchers have mainly focused on improving their formability by adding alloying elements, but studies have paid little attention to the effect of thermomechanical processing conditions on formability [20,21,22]. For example, the synergetic additions of low-cost Al and Mn elements in Mg–6Zn–0.2Ca form a fine grain structure and a unique texture feature, which contributes to a good Erichsen value of 7.2 mm [21]. More studies that analyze the relationship between different rolling parameters and formability need to be conducted. Therefore, in this paper, we used three different rolling parameters to prepare Mg–2Zn–0.5Ca (wt.%) alloy sheets, and systematically investigated these sheets’ microstructures, textures, and formability at room temperature. The study aims to provide guidance for the preparation of Mg–Zn–Ca alloy plates with high formability.

## 2. Materials and Methods

In this study, the cast ZX20 (Mg–2 wt.% Zn–0.5 wt.% Ca) alloy was used as the initial material. First, 5 mm thick plates were cut from the ingot, and these were homogenized at 360 °C for 24 h, followed by water quenching. The rolling test was carried out on an LG-300 two-roll roller machine (made in China by China first heavy industries) with a maximum rolling force of 30 t, which has a roll diameter of 170 mm and a roll width of 300 mm. The rolling speed was 0.2 m/s. The homogenized sheets were then rolled to 4 mm at 360 °C for 4 passes using cross-rolling (CR), with a reduction of ~5% per pass (~0.25 mm thickness reduction per pass) occurring. For cross-rolled sheets, the direction of the first pass of rolling is defined as the rolling direction. The cross-rolled sheets were annealed at 360 °C for 40 min and then were rolled along the rolling direction to a final thickness of 0.8 mm, with a total 80% reduction under the three different rolling conditions taking place: (1) Some sheets were rolled at 300 °C for 8 passes with a pass rolling reduction of 10~33% (~0.4 mm thickness reduction per pass). (2) Some sheets were rolled at 360 °C for 8 passes with a pass rolling reduction of 10~33% (~0.4 mm thickness reduction per pass). (3) Some sheets were rolled at 360 °C for 4 passes with a pass rolling reduction of 20~50% (~0.8 mm thickness reduction per pass). The sheets, between passes, were reheated at the rolling temperature for 10 min. After rolling, the sheets were annealed at 360 °C for 30 min to obtain better comprehensive mechanical properties. The detailed rolling parameters for preparing R1, R2, and R3 sheets are listed in Table 1.

Samples for microstructure observation were first polished with sandpaper with numbers from 400# to 3000# and diamond spray, and then the polished samples were etched with a picric acid solution. The microstructures of the samples were observed by optical microscopy (OM, ZEISS-AXIO Scope A1 produced by Carl, Zeiss, Germany) and scanning electron microscopy (SEM, FEI Nova 400 FEG-SEM made in America by FEI company). An X-ray diffractometer (XRD Rigaku, D/max-2500PC made in Japan by Rigaku Corporation) equipped with a Cu radiation source (λ = 0.15406 nm) was used to characterize the macroscopic textures of the samples. Four incomplete pole figures of (0002), (10–10), (10–11), and (10–12) were measured by the Schulz reflection method in the range of α from 0 to 70° and β from 0 to 360°, and Labotex software was used to calculate the grain orientation distribution function and to calculate the complete pole figure. The EBSD samples were prepared using sandpaper grinding and electrochemical polishing at 20 V for 90 s in ACII polishing solution. Then, Electron backscatter diffraction (EBSD) was performed on TESCAN MIRA3 (Tescan Corporation, Brno, Czech Republic) with the HKL-EBSD system. The step size for EBSD scanning was from 1.2 to 2 μm based on microstructure.

Dog-bone-shaped tensile specimens 14 mm in length and 4 mm in width were ma-chined from the annealed plates along the rolling direction (RD), 45°, and along the transverse direction (TD). Tensile tests were carried out at room temperature on a Shimadzu universal tensile testing machine using a strain rate of 1 × 10^−3^ s^−1^. For each direction, the tensile test was repeated three times and the average value was taken at the end. Moreover, an extensometer with two clips was used to measure strain along the tensile direction, and Lankford values (r-value) were examined at 10% nominal tensile strain. To evaluate the stretch formability of the annealed sheets, 50 × 50 mm square samples were cut from the three sheets and subjected to Erichsen cupping tests using a 20 mm diameter hemispherical punch at room temperature. The punch speed and the blank holder force were 3 mm/min and 10 kN, respectively. The Erichsen cupping test was repeated three times for each sheet to exclude errors in individual samples.

## 3. Results

### 3.1. Microstructure and Texture Evolution

**Figure 1:**
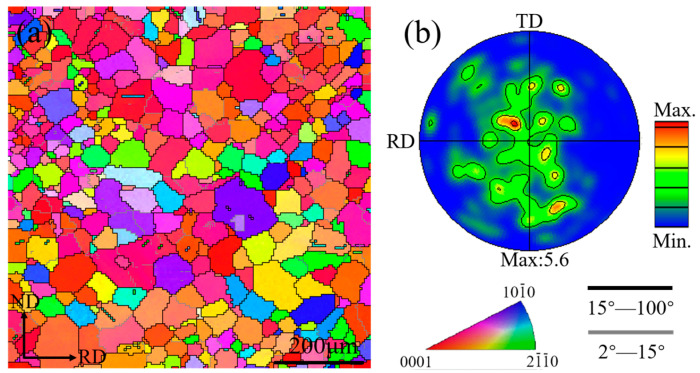
(**a**) EBSD inverse pole figure maps (IPF) and (**b**) (0002) pole figures of ZX20 alloy subjected to cross-rolling and annealing.

The inverse pole figure maps and (0002) pole figures of the sheet after cross-rolling and annealing are shown in Figure 1. The average grain size of the cross-rolled sheet was measured to be 38.2 μm. The sheet exhibited an inhomogeneous microstructural feature consisting of coarse and fine grains. In the (0002) basal pole figure, the grains were distributed along RD and TD, which indicated that the grain orientation can be modified by CR.

**Figure 2:**
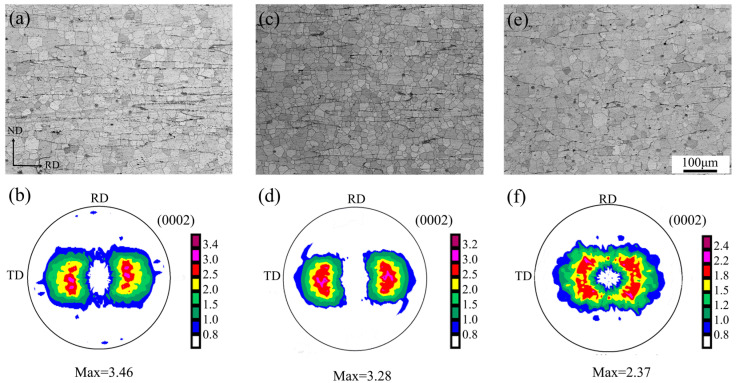
Optical micrographs and (0002) pole figure of ZX20 alloy plates prepared by different rolling parameters after annealing: (**a**,**b**) R1, (**c**,**d**) R2, and (**e**,**f**) R3.

Figure 2 shows the optical microstructures and (0002) basal texture measured by the XRD of the plates prepared under different rolling conditions after they were annealed. The sheets had an equiaxed grain structure and had been fully recrystallized after their annealing at 360 °C for 30 min. Further, a large number of second-phase particles were also observed in the microstructures of the sheets. This suggests that the second phases were not able to be completely dissolved into the matrix. The maximum intensity of the (0002) basal pole of the sheets produced by the three different rolling conditions was located at TD, where the R1 sheet had the highest basal pole intensity at 3.46 m.r.d., the R3 sheet had the weakest basal pole intensity at 2.37 m.r.d., and the R2 sheet had a moderate basal pole intensity of 3.28 m.r.d. The basal pole intensity of the R3 sheet was much lower than that of the R1 and R2 sheets. It is shown that the intensity of basal texture can be reduced by increasing rolling temperature and/or increasing the single-pass rolling reduction. The angular distribution of the basal pole was considerably more broadened along the TD than along the RD in the three sheets. Furthermore, the basal pole distributions of the three sheets exhibited different features in the plane. The R2 sheet had a bimodal texture, with its basal pole being tilted away from the normal direction (ND) toward TD. In the R2 sheet, the vast majority of grains were distributed along TD, and only a very small portion of grains were tilted away from ND toward RD. However, the R1 and R3 sheets developed an elliptical texture, i.e., their basal poles were not only tilted from ND toward TD by about 40° but they were also tilted from ND toward RD by about 20°. There were also some differences in grain orientation distribution along RD in R1 and R3 sheets. Compared to R1, in the R3 sheet, a larger number of grains were distributed along RD and the angular distribution of the basal pole was also wider than that in the R1 sheet.

**Figure 3:**
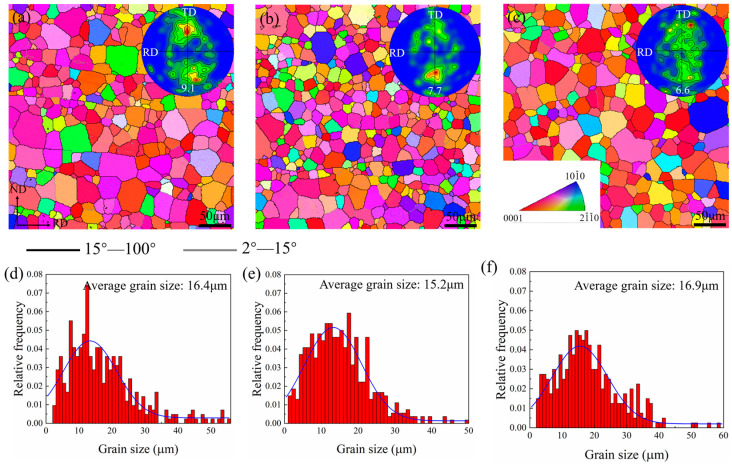
EBSD inverse pole figure maps (IPF), (0002) pole figures, and grain size distribution maps reflecting the microstructures and micro-textures of (**a**,**d**) R1, (**b**,**e**) R2, and (**c**,**f**) R3 annealed sheets.

As seen in Figure 3, the microstructures of the sheets were further characterized by EBSD. The inverse pole figure maps (IPF) show that the three sheets prepared by different rolling processes were fully recrystallized after they were annealed. The (0002) pole figure measured by EBSD was in agreement with the macro-texture measured by XRD. The grain size distributions of the sheets are shown in Figure 3d–f. The microstructure of the cross-rolled sheets achieved significant refinement after being rolled under different rolling conditions. The average grain sizes were 16.4 μm for the R1 sheet, 15.2 μm for the R1 sheet, and 16.9 μm for the R1 sheet. Across the three sheets, their grain sizes were concentrated within the range of 10~20 μm, which shows that the sheets had similar homogeneous microstructures.

**Figure 4:**
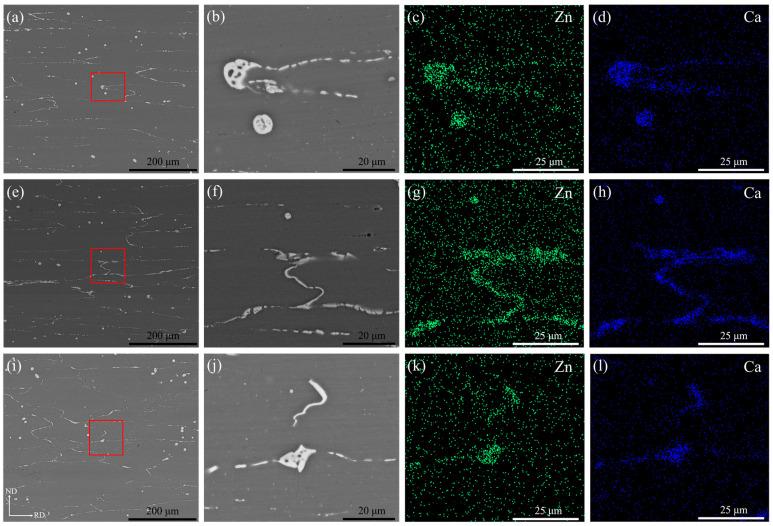
Backscattered electron (BSE) images and energy dispersive X-ray spectroscopy (EDS) maps of as-annealed ZX20 plates under different rolling parameters (**a**–**d**) R1, (**e**–**h**)R2, and (**i**–**l**) R3. Note that (**b**), (**f**) and (**j**) are high magnification images taken from red rectangles in (**a**), (**e**) and (**i**), respectively.

Figure 4 shows backscattered electron (BSE) images and energy dispersive X-ray spectroscopy (EDS) maps of the (a–d) R1, (e–h) R2 and (i–l) R3 as-annealed sheets. After annealing was performed, a large number of second-phase particles remained in the three sheets. Moreover, the second phase particles were obviously broken into small particles distributed along the grain boundary during rolling. According to the BSE images, it can be noticed that the area fraction of the second phase in the R3 sheet is relatively lower compared to that in the R1 and R2 sheets. For the R3 sheet, the large rolling reduction per pass was more favorable for the second phase to break into small particles, which may be dissolved into the matrix during annealing between passes. Among the three sheets only one type of second phase, enriched with Zn and Ca atoms, was present. In previous studies on the Mg–Zn–Ca alloy, it was suggested that Ca has a maximum solid solution of 0.2 wt.% at room temperature in magnesium alloys and that Ca solid solubility decreases with increasing Zn content [23,24]. Moreover, researchers have found a large amount of insoluble Ca_2_Mg_6_Zn_3_ phase in the Mg–Zn–Ca alloy [25,26]. Therefore, it can be confirmed that in this study, the second phase was Ca_2_Mg_6_Zn_3_.

### 3.2. Tensile Properties

**Figure 5:**
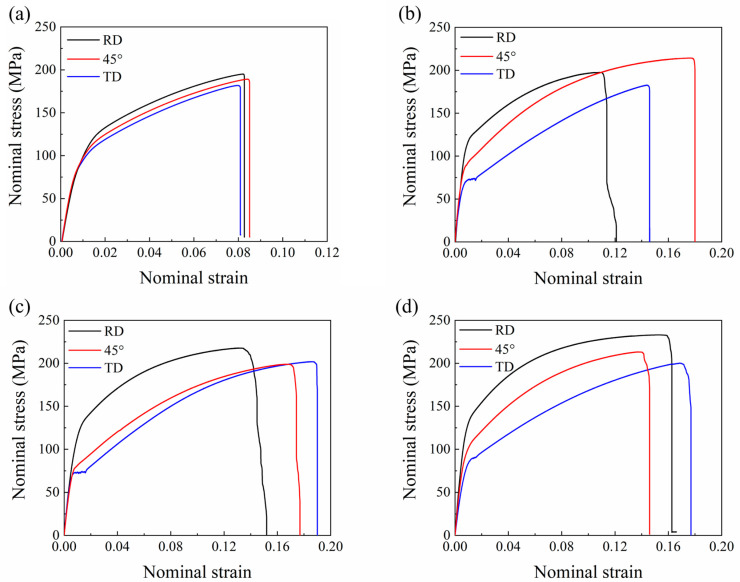
Nominal tensile stress–strain curves for annealed (**a**) CR, (**b**) R1, (**c**) R2, and (**d**) R3 samples along the RD, 45°, and TD.

Figure 5 shows the typical nominal stress vs. strain curves from the tensile experiments along the RD, 45°, and TD directions for the (a) CR, (b) R1, (c) R2, and (d) R3 samples, respectively. The calculation of the nominal stress–strain curve is the same as the engineering stress–strain curve. Tensile and mechanical anisotropy data for the three samples, including ultimate tensile strength (UTS), yield strength (YS), and elongation to failure (EF) data, are summarized in Table 2. The EF of the sheets prepared with three different rolling parameters showed a significant increase compared to the cross-rolled sheets, which could be attributed to the grain refinement. Generally, the YS and UTS along RD were higher than those along TD, but the opposite was the case for the EF. From the grain orientation distribution in Figure 2 and Figure 3, it is known that the grain basal pole had a broader distribution along TD than that along RD. Therefore, when tensile along RD and TD, respectively, the grains along TD have a higher Schmid factor, thus leading to a lower YS along TD. In the three samples, the YS of R1 and R2 were almost equal along RD and TD, and both were lower than that of R3 in the same direction; especially the YS of R3 along TD increased obviously. Meanwhile, the EF along the RD increased from 11.2% and 14.3% for R1 and R2 to 16.2% for R3. The curves of R1 and R2 along TD show a flat stress after yielding. This may be influenced by twinning and the second phase. In particular, R1 exhibited the highest UTS and EF in the 45° direction. The increase in EF along 45° may be related to the different distribution of texture and the different characteristics of the second phase caused by the rolling parameters. Due to the increase in plasticity along 45°, the sheet exhibited a stronger work hardening which finally showed the highest UTS in R1. In addition, Table 2 compares the Lankford values (r-value), average r values (r_avg_), and planar anisotropy (Δr) for R1, R2, and R3, respectively. The r value was calculated with equation r = ε_w_/ε_t_, where ε_w_ and ε_t_ are the true strains along the width and thickness directions, respectively, at 10% tensile deformation. The three sheets exhibited different mechanical anisotropy behaviors. R1 and R3 exhibited lower average r values and plastic anisotropy than R2.

### 3.3. Stretch Formability

**Figure 6:**
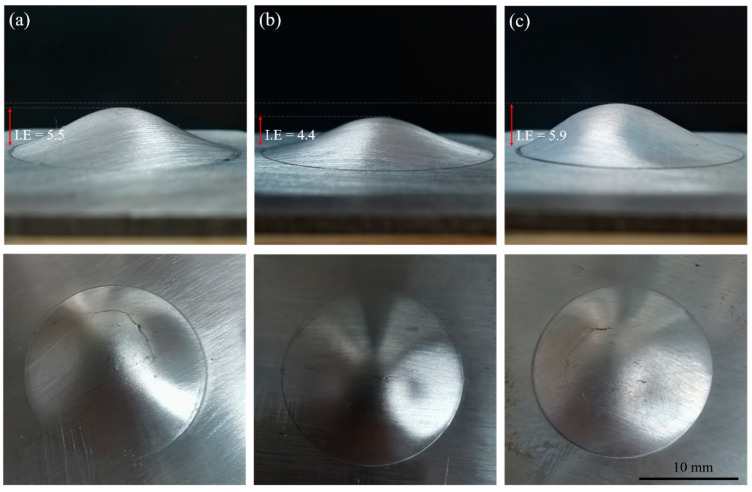
The typical appearance of (**a**) R1, (**b**) R2, and (**c**) R3 annealing plates. These images were taken after the Erichsen cupping test at room temperature.

The stretch formability of the (a) R1, (b) R2, and (c) R3 samples at room temperature was evaluated by the Erichsen cupping test, as shown in Figure 6. The index Erichsen (I.E.) values for R1, R2. and R3 were 5.5 mm, 4.4 mm, and 5.9 mm, respectively. Further, the stretch formability of R3 was better than that of R2 by 34%, indicating that its secondary formability was enhanced. Significant differences in I.E. values were observed for the three sheets prepared according to different rolling parameters. Some studies have shown that the formability of a sheet is strongly dependent on its microstructure, texture, and plastic deformation mechanism during stretch forming [17,27]. Therefore, in this study, it was necessary to systematically investigate the three sheets’ microstructures and texture evolution as well as their plastic deformation mechanisms during stretch forming.

**Figure 7:**
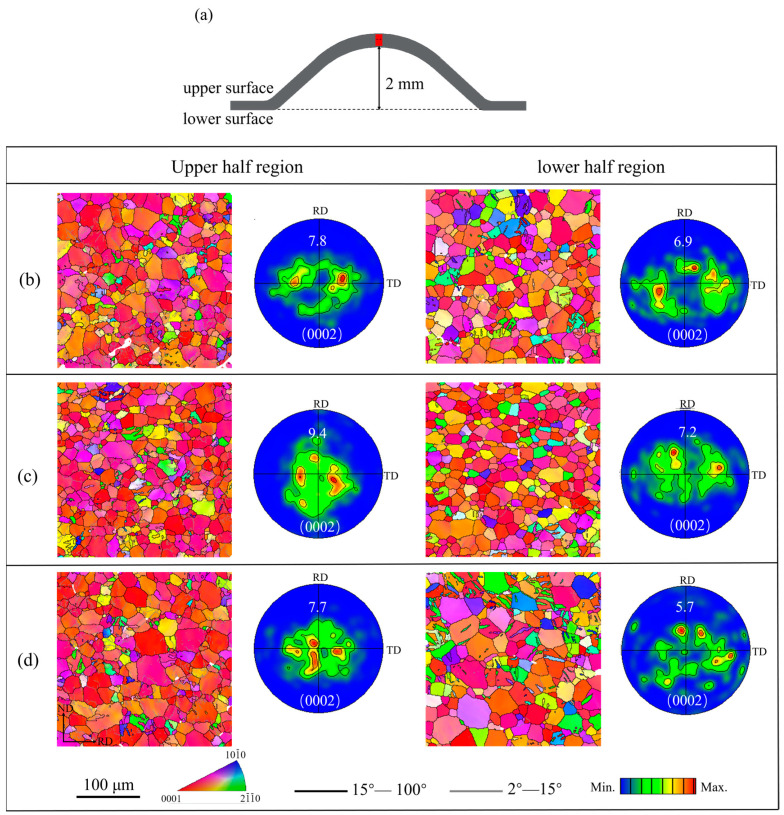
(**a**) Schematic drawings of the microstructure characterization of the cupping test, EBSD IPF maps, and (0002) pole figures of (**b**) R1, (**c**) R2, and (**d**) R3 samples after Erichsen cupping test to 2 mm.

**Figure 8:**
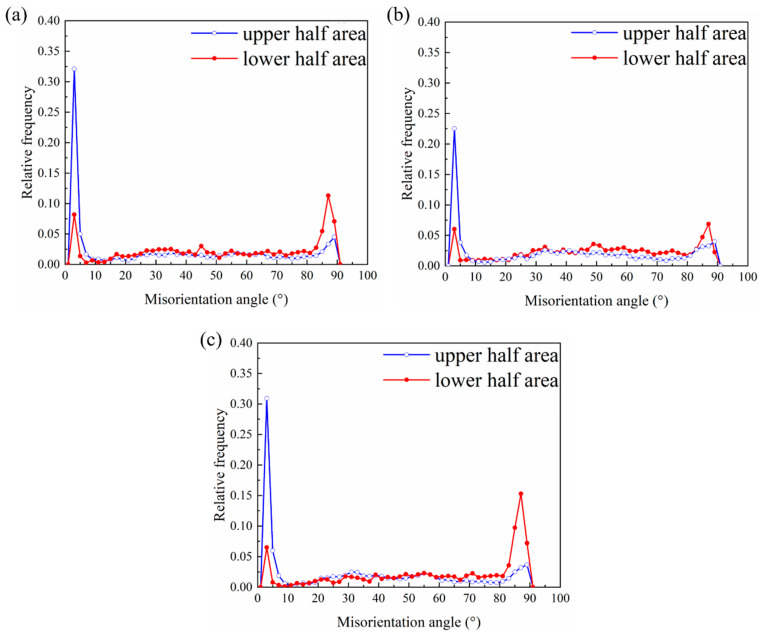
Distributions of misorientation angle profiles at different positions in the top areas of (**a**) R1, (**b**) R2, and (**c**) R3 after stretch forming to a height of 2 mm.

To understand the high formability mechanism of the R3 sample, the evolution of its microstructure and texture during stretch forming was analyzed via the EBSD technique. The EBSD data acquisition positions are shown in Figure 7a, and the position is close to the upper/lower surface. The IPF maps and (0002) pole figures of the three samples after the Erichsen cupping test to a 2 mm height are shown in Figure 7b–d. After 2 mm stretch forming, the angular distribution of the basal poles became distinctly narrower from RD and TD to ND compared to the annealed (0002) pole figure. Additionally, the (0002) basal texture intensities in the upper half region were stronger than those in the lower half region. It should be noted that the intensities of the basal poles in the corresponding region of R3 were the weakest among the three samples (Figure 7d). Figure 8 shows the misorientation angle distributions in the upper half region and lower half region for the 2 mm stretch-formed samples. As a sharp peak occurred near 86°, it is clear that a large number of tension twins were activated during the early stretch forming due to the fact that the {10–12} tension twins are intimately related to the misorientation angle approaching 86° [28,29]. The twinning fraction was the highest in the R3 sample and the lowest in the R2 sample. Meanwhile, the twin fraction was moderate in the R1 sample. This result was in agreement with the Erichsen cupping values of the sheets. From the distribution of the misorientation angles in Figure 8, a different characteristic of the misorientation angle between the upper and lower half areas of the samples after 2 mm stretch forming can be observed. The peak misorientation angle in the lower half region was near 86°, but the peak misorientation angle in the upper region was in the range of 1~5°. Further, sharp increases in the fraction of low-angle grain boundaries (LAGB) suggest that the initiation of dislocation slip deformation in the upper region occurred. This slip deformation was found to be of importance to the formability during stretch forming, especially to the formability of the non-basal slip. 

**Figure 9:**
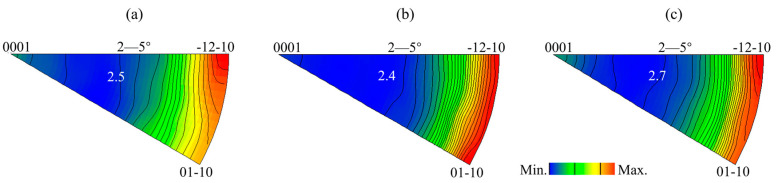
Distributions of in-grain misorientation axes (IGMA) of (**a**) R1, (**b**) R2, and (**c**) R3 after stretch forming to 2 mm in the upper top region.

To determine whether non-basal slip was involved in the deformation during stretch forming, the in-grain misorientation axes (IGMA) were adopted to distinguish between the kinds of dislocations that were activated [30,31,32,33]. In this study, the misorientation angle of 2~5° of the grain was used to analyze the deformation mechanism during stretch forming, as shown in Figure 9 [34]. Across the three samples, the IGMA mainly lay along the [01–10]~[−12–10] arc, supporting those LAGBs that had a misorientation axis of [uvt0]. On the other hand, some IGMA were found to be lying along [0001], suggesting that there were LAGBs with a misorientation axis of [0001] in the R1 and R3 samples. However, no IGMA distribution along [0001] was found in the R2 sample. According to the IGMA analysis, it is likely that both basal <a> and pyramidal <c + a> slips were activated when the IGMA were lying between [01–10] and [−12–10]. The IGMA lying along [0001] can only be activated by prismatic <a> dislocations [31,35]. As the critical resolved shear stress (CRSS) for the pyramidal <c + a> slip was much higher than that for the basal <a> slip, it is difficult to initiate this at room temperature [36,37]. Thus, the effect of the pyramidal <c + a> slip on the formability at room temperature may have been negligible. Compared to the R2 sample, in addition to the basal <a> slip, the prismatic <a> slip was also involved in the deformation of the R3 sample. Therefore, it can be concluded that the high formability of the R3 sample was facilitated by the activation of the prismatic <a> slip, in addition to tensile twins.

## 4. Discussion

In this study, the microstructures, textures, and mechanical properties of the three sheets (R1, R2, and R3) were examined in detail, in particular their stretch formability at room temperature. R3 displays the highest I.E. value of 5.9 mm. As seen in Table 2, the R2 sheet, which had a high $\bar{r}$-value, exhibits low stretch formability. Meanwhile, the R3 sheet, which had a low $\bar{r}$-value, exhibited high stretch formability. Previous studies have also focused on the relationship between the $\bar{r}$-value and formability [27,38]. It is believed that the $\bar{r}$-value is inversely related to formability [38]. The $\bar{r}$-value being associated with deformation through the thickness direction indicates that the smaller the $\bar{r}$-value, the greater the strain through the thickness direction, i.e., the enhancement of the thickness thinning ability of the sheet during stretch forming contributes to improvements in the sheet’s formability. In this paper, the $\bar{r}$-values are also negatively correlated with the I.E. value. This means that the strain in the thickness direction is greater in the R3 sheet than that in the R2 sheet during stretch forming. r values are calculated involving strains in both width and thickness, so they are closely related to texture. The basal pole of grains in both the R1 and R3 sheets were tilted away from ND toward RD, forming an elliptical texture. However, the vast majority of the basal pole in the R2 sheet was tilted toward TD, forming a bimodal texture along TD that was unfavorable to the thickness thinning of RD. Therefore, the ability of thickness deformation in the R1 and R3 was stronger than in the R2 sheet. Moreover, the R3 sheet had the weakest texture intensity compared to the R1 and R2 sheets. This also favored the R3 sheet to exhibit the highest I.E. value.

It has recently been noted that the {10–12} tension twins play a crucial role in the stretch formability of sheets, and they have been verified to enhance formability in magnesium alloys [30,39]. The activation of tension twins increases the average Schmid factor for the basal slip in the twinning area, resulting in the high formability of the Mg–0.4Sn–0.7Y–0.6Zn alloy sheet [38]. This shows that twinning converts a hard orientation grain to a soft orientation grain, which is favorable for deformation, and it also accommodates plastic deformation [40,41]. During the stretch-forming process, there are opposite stress states applied to the upper and lower half regions of the sheet. The upper half region of the sheet is dominated by tensile stresses, and the lower half region is dominated by compressive stresses [39]. Twinning can be activated through two methods: one involves creating tension parallel to the c-axis, and the other involves creating compression perpendicular to the c-axis [42]. In the biaxial stress state, the grains with basal poles close to the RD or TD will tend to twin on the upper half region of the sheet due to tensile stress. In the lower half region, the grains with basal poles parallel to ND are subjected to twinning by compressive stress. This suggests that the grain orientation distribution plays a decisive role in the twinning fraction. In this study, the R3 sample developed a broader basal pole angular distribution along both RD and TD than that of the R2 sample. Therefore, R3′s grain orientation was more favorable for twinning, which led to a higher twinning fraction, whose ability to accommodate deformation was improved. In the R2 sample, the vast majority of grains were distributed along TD, which led to a significant reduction in twinning fraction during stretch forming. This also implies that the ability of twinning to accommodate deformation was weaker in the R2 sample. However, compared with the R2 sample, the R1 sample had more grains tilted along RD, which was more favorable for twinning than R2 during stretch forming, so the R1 sample exhibited a higher I.E. value than the R2 sample. Ultimately, the R3 sample achieved the highest formability.

The largest I.E. value was obtained for the R3 sample, owing to its weaker texture intensity (2.37 m.r.d.) and its elliptical texture. The weakening of texture allows for a majority of grains to be oriented favorably to the basal slip. Therefore, the R3 sample exhibited the highest I.E. value. On the contrary, the R2 sample had relatively lower I.E. values due to the higher intensity of (0002) basal texture resulting in insufficient grains to provide slip deformation. This suggests that the improved formability of the R3 sample was associated with a weaker texture. Furthermore, the R3 sample developed an elliptical texture after it was annealed, with its grain basal poles simultaneously being tilted 20° away from ND toward RD and 40° toward TD. This kind of texture has been reported in previous studies [11,20,22]. Further, a broadened distribution of basal poles along both RD and TD contributes to a basal <a> slip and {10–12} extension twinning during stretch forming, and also leads to a higher I.E. value [39]. IGMA measurements confirmed that the prismatic <a> slip was activated in the R3 sample. Although the basal slip is the dominant plastic deformation mechanism during stretch forming, the non-basal dislocation slip accommodates deformation along the thickness direction and plays a positive role in enhancing formability. Bian et al. have argued that the activation of the prismatic <a> slip is crucial for Mg–1.2Al–0.3Ca–0.4Mn–0.3Zn alloy to achieve high formability [13]. Further, researchers have noticed that the addition of a small amount of Ca to AZ31 alloy facilitates the enhancement of its stretching formability by activating the non-basal slip [14]. Consequently, the improvement in the formability of the R3 sample was closely related to its grain orientation distribution and the weakening of its texture. The study also demonstrated that the texture of ZX20 alloy can be modified by adopting a reasonable combination of thermomechanical processing in order to achieve more desirable formability. In the next step, we will adopt the optimal thermomechanical processing parameters in this paper to investigate the formability of the alloy with different Ca concentrations to understand the effect of Ca concentration on the formability, so that the composition of the ZX-system alloy can be optimized. The work in this paper provides the foundation for the next step of the experiment.

## 5. Conclusions

In this study, the effect of different rolling parameters on the stretch formability of Mg–2Zn–0.5Ca (wt.%) alloy was investigated. A sheet rolled at 360 °C with a pass rolling reduction of 20~50% exhibited the highest I.E value of 5.9 mm. Sheets rolled at 360 °C and 300 °C with a pass rolling reduction of 10~33% showed, respectively, I.E. values of 4.4 mm and 5.5 mm. The I.E. value of the former was improved by 34%. For the sheet rolled at 360 °C with a pass rolling reduction of 20~50%, the angular distribution of the basal poles was broadened along RD and TD; further, an elliptical texture developed, with the sheet’s basal poles tilting away from ND towards RD by nearly 20° and also towards TD by about 40°. Additionally, the maximum intensity of this sheet’s basal poles was 2.37 m.r.d., which was the smallest among the three sheets. Microstructural characterization during stretch forming shows that in addition to the basal <a> slip, the prismatic <a> slip and tension twins were also activated. Additionally, compared with the sheet rolled at 360 °C with a pass rolling reduction of 10~33%, the prismatic <a> slip was quite active and the activity of the tension twins was much higher in the sheet rolled at 360 °C with a pass rolling reduction of 20~50%. Therefore, the enhancement of formability can be ascribed to this sheet’s weakened (0002) basal texture and its development of an elliptical texture.

## Figures and Tables

**Table 1:** Detailed parameters of the different rolling processes in this study.

Samples	Temperature (°C)	Pass Rolling Reduction (%)	Thickness Reduction per Pass (mm)	Total Reduction (%)	Total Passes
R1	300	10~33	0.4	80	8
R2	360	10~33	0.4	80	8
R3	360	20~50	0.8	80	4

**Table 2:** Room-temperature tensile mechanical properties of ZX20 alloy plates along RD, TD, and 45° under different rolling conditions.

Sample	Direction	UTS/MPa	YS/MPa	EF/%	r-Value	r_avg_	Δr
CR *	RD	195.3 ± 2.8	106.3 ± 0.8	8.2 ± 0.1	0.86	0.88	0.09
	45°	188.8 ± 1.6	101.9 ± 1.2	8.5 ± 0.2	0.93		
	TD	182.0 ± 3.7	97.6 ± 0.5	8.1 ± 0.2	0.82		
R1	RD	197.6 ± 2.7	120.5 ± 1.6	11.2 ± 0.2	0.61	0.60	0.13
	45°	215.1 ± 5.8	90.9 ± 2.1	18.0 ± 0.4	0.67		
	TD	183.6 ± 4.2	72.3 ± 0.3	14.6 ± 0.1	0.45		
R2	RD	218.3 ± 2.4	127.4 ± 3.9	14.3 ± 0.3	0.63	0.68	0.22
	45°	202.6 ± 3.1	80.0 ± 0.9	17.4 ± 0.2	0.80		
	TD	199.7 ± 1.1	72.4 ± 0.5	18.9 ± 0.2	0.52		
R3	RD	233.3 ± 4.3	137.5 ± 4.7	16.2 ± 0.1	0.81	0.62	0.14
	45°	213.9 ± 2.8	101.9 ± 1.8	14.5 ± 0.2	0.55		
	TD	200.6 ± 1.7	89.0 ± 0.6	17.6 ± 0.1	0.57		

* Since the CR sheet failed before 10% strain, the r-value measurements of the CR sheet were performed on the failed samples.

## Data Availability

Data are contained within the article.
